# α-Tocopherol Ameliorates Redox Equilibrium Disorders and Reduces Inflammatory Response Caused by Diclofenac-Induced Nephrotoxicity in Male Wistar Rats

**DOI:** 10.7759/cureus.50474

**Published:** 2023-12-13

**Authors:** Samuel Dada, Temitope Fabiyi-Edebor, Olabode Akintoye, Okechukwu Ezekpo, Oluwasina Dada, Titilope Bamikefa, Joseph Sanya

**Affiliations:** 1 Department of Human Physiology, College of Medicine and Health Sciences, Afe Babalola University, Ado-Ekiti, NGA; 2 Nephrology Unit, Department of Medicine, Ekiti State University Teaching Hospital/ Ekiti State University, Ado-Ekiti, NGA; 3 Department of Human Physiology, College of Medicine, Ekiti State University, Ado-Ekiti, NGA; 4 Renal Surgery Department, Queen Elizabeth Hospital, Birmingham, GBR; 5 Department of Medicine, College of Health Science Osun State University, Osogbo, NGA

**Keywords:** nonsteroidal anti-inflammatory drugs, vitamin e, nephrotoxicity, oxidative stress, cytokines

## Abstract

Background

Diclofenac (DCF), a nonsteroidal anti-inflammatory drug (NSAID), is widely used for its analgesic and anti-inflammatory properties, but it can also be nephrotoxic. Vitamin E (α-tocopherol) has been shown to protect against renal toxicity caused by various agents, including NSAIDs. This study aims to evaluate the pathophysiology of renal damage and the nephroprotective effect of vitamin E against DCF-induced renal damage in male Wistar rats.

Animal and methods

Twenty-four male Wistar rats, divided into six equal groups, were used for the study. Group 1 (control group) was treated with distilled water only, while the other groups received either high or low doses of DCF with or without a fixed dose of vitamin E. Renal function was assessed by measuring serum urea, creatinine, and kidney injury molecule-1 (KIM-1). Oxidative damage and renal antioxidant levels were also assessed. Additionally, the expression of nuclear factor kappa-light-chain-enhancer of activated B cells (NF-κB), renal cytokine tumor necrosis factor-α (TNF-α), and histopathological changes were evaluated.

Results

DCF caused a significant increase in serum urea, creatinine, KIM-1, TNF-α, NF-κB, and malondialdehyde levels compared to the control group. However, in the groups treated with DCF plus vitamin E, a significant reduction (P<0.05) in the levels of pro-inflammatory cytokines and malondialdehyde was observed, along with improvement in renal function indices, superoxide dismutase, catalase, and glutathione peroxidase levels comparable to the control group. The observed renal histopathological changes were consistent with the results of the biochemical parameters between the treated groups and the normal control rats.

Conclusion

Findings from this investigation suggested that DCF can be nephrotoxic at a certain dose when used for a prolonged duration. Co-administration of vitamin E suppressed the elevated inflammatory cytokines and led to changes in the cell redox-sensitive signaling pathways induced by DCF, with eventual amelioration of the nephrotoxicity.

## Introduction

Nonsteroidal anti-inflammatory drugs (NSAIDs), such as diclofenac (DCF), are a class of drugs commonly used for their analgesic and anti-inflammatory effects [1]. In the medical literature, the use of DCF has been associated with different forms of kidney damage in both experimental animals and humans. Oxidative stress has been shown to contribute to the pathophysiological mechanism of kidney injury resulting from NSAID ingestion [1,2].

In numerous animal models of kidney disease, the administration of various natural or synthetic antioxidants has shown a beneficial effect in preventing and ameliorating renal damage [3-5]. Emerging evidence suggests that vitamins with antioxidant properties, such as vitamin E (α-tocopherol), may have therapeutic potential to halt or slow the decline in kidney function in DCF-induced nephrotoxicity [6]. However, available data on the nephroprotective function of vitamin E against DCF-induced kidney damage are sparse and inconsistent. Therefore, this study aims to evaluate the nephroprotective effect of vitamin E (α-tocopherol) against DCF-induced kidney damage by assessing biochemical markers of renal dysfunction, oxidative stress, and renal histological changes in male Wistar rats.

## Materials and methods

Animals

Twenty-four healthy male Wistar rats weighing between 200 and 220 grams (11-12 weeks old) were used in the study. They were obtained from the animal house of the College of Medicine, Ekiti State University, Ado-Ekiti, Nigeria. The rats were randomly divided into six equal groups, housed individually in plastic cages, and acclimatized for a week. Standard laboratory conditions, including a natural light/dark cycle, room temperature, and humidity, were maintained, and the rats were fed standard rat pellets and distilled water ad libitum. Ethical clearance was obtained from the Ethical Committee of the College of Medicine, Ekiti State University (EKSU/A67/2022/02/021). The rats were handled per the National Research Council's Guide for the Care and Use of Laboratory Animals [7].

Drugs and vehicle

All drugs were purchased from a commercial pharmacy. Vitamins E (alpha-tocopherol acetate) ENAT 400 IU soft gel capsules, a natural vitamin E product containing 285 mg per capsule, were manufactured by Mega Lifesciences Public Company Limited, Samutpakarn, Thailand. The DCF potassium tablets (Cataflam® 50 mg by Novartis, Basel, Switzerland) were dissolved in distilled water and administered orally to rats using rat cannulas according to the study protocol. The dose of vitamin E used in this study was based on published studies showing that this concentration is effective in reducing lipid peroxidation and oxidative kidney damage in experimental animal models [4,6,8]. Similarly, the dose of DCF that was sufficient to cause renal damage was based on previous reports [2,9,10]. The drugs were administered orally for 10 days according to the study protocol.

Experimental design

First group: (control group) distilled water; second group: high-dose (30 mg/kg) DCF only, third group: vitamin E only (250 mg/kg), fourth group: high-dose (30 mg/kg) DCF, plus vitamin E (250 mg/kg orally); fifth group: low-dose (10 mg/kg) DCF only; and sixth group: low-dose (10 mg/kg) DCF, plus vitamin E (250 mg/kg) orally.

Collection of tissue and blood samples for biochemical analysis

At the end of the experiment, the rats were sacrificed by the cervical dislocation method. Whole blood samples were collected from each rat using a cardiac puncture technique with sterile needles and syringes into clean anticoagulant-free bottles. The blood was allowed to coagulate for about 30 minutes, and then the serum in each tube was separated by a cold centrifuge at 4000 rpm for 10 minutes to obtain sera. The serum was collected with the aid of a Pasteur pipette into plain bottles and kept on ice for analysis. Both kidneys were harvested immediately and rinsed in ice-cold physiological saline.

Preparation of kidney homogenates and histology specimen

The left kidney was transferred into a plain bottle containing 10% buffered formalin for histology, while 10% of the right kidney tissue from each rat was homogenized in 100 mM potassium phosphate buffer at pH 7.4 using a mortar and pestle and then centrifuged at 10,000 rpm for 10 minutes at 4 °C in a cold centrifuge. The supernatant was collected and used to determine the oxidative stress and antioxidant markers (malondialdehyde (MDA), kidney catalase, glutathione peroxidase, and superoxide dismutase (SOD) activities). ELISA kits (Fortress Diagnostics, Antrim, Ireland) were used for the determination of proinflammatory markers (NF‑κB-P65), kidney cytokines (TNF-α), and KIM-1 according to the manufacturer's instructions.

Serum biochemistry

Serum creatinine was analyzed using the method described by Bartels et al. [11] based on the principle that creatinine in an alkaline solution reacts with picric acid to form a colored complex. The amount of the complex formed is directly proportional to the creatinine concentration as read spectrophotometrically at 490 nm, and the serum urea was measured using the method used by Veniamin et al. [12].

Measurement of enzyme activities in renal homogenates

Catalase Activity

Kidney catalase activity was assayed using Beers et al.'s method [13]. A blank containing potassium phosphate buffer was used. Moreover, 20 µL of kidney supernatant sample and 200 µL of hydrogen peroxide were added, and the decrease in absorbance at 240 nm after the addition of the substrate was followed spectrophotometrically.

SOD

Kidney SOD activity was measured using the method of Misra et al. [14], which measures the inhibition of the rate of reduction of cytochrome c by the superoxide radical, observed at 550 nm. A mixture of 0.5 mL of tissue homogenate and 0.05 M carbonate buffer (pH 10.2) with distilled water added to equilibrate was placed in a spectrophotometric cuvette. The reaction was started by adding 0.3 mL of substrate 0.3 mM epinephrine. The increase in absorbance at 480 nm was monitored every 30 seconds for 150 seconds. The kidney SOD activity was expressed in mmol/min/mg protein.

Glutathione Peroxidase

Fortress kit (Fortress Diagnostic Limited, United Kingdom) was used for the quantitative determination of the total glutathione peroxidase (GPx) according to the manufacturer’s instructions.

Measurement of Lipid Peroxidation Levels

MDA

The concentration of MDA was quantified according to the method of Buege et al. [15]. An aliquot of kidney homogenate (0.4 mL) was mixed with Tris-KCl buffer (1.6 mL) containing 0.5 mL of trichloroacetic acid (30%). The mixture was thoroughly heated in a water bath at 1000 C for 45 minutes. It was then cooled and centrifuged at 4000 rpm for 10 minutes. The absorbance of the supernatant was read at a wavelength of 540 nm against a reference blank of distilled water after centrifuging for another 10 minutes. The lipid peroxidase activity was expressed in µmol/ml.

Histopathological Examination of the Renal Tissue

The left kidney tissue samples were fixed in 10% buffered formalin for 48 hours, dehydrated in 95% ethanol, cleared in xylene, and embedded in paraffin wax. Sections of the kidney tissue were prepared and stained with H&E. The prepared slides were examined under an OPTO-Edu industrial camera light microscope, and photomicrographs were taken.

Statistical analysis

The data obtained were entered into and analyzed with GraphPad Prism 6.01 (GraphPad Software, Inc., La Jolla, CA). All measured parameters were presented as mean ± SD and analyzed using one-way ANOVA, followed by Tukey's HSD test. The P values of less than or equal to 0.05 were considered statistically significant.

## Results

Effect of treatment on kidney function

**Figure 1:**
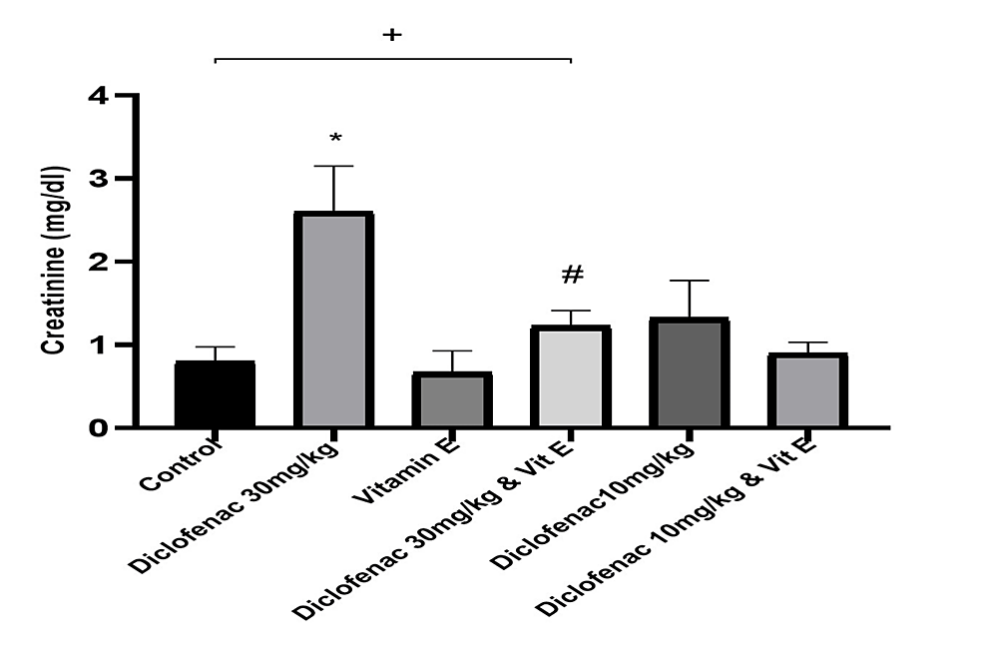
Effect of treatments on the level of serum creatinine Data are expressed as mean ± SD. (n = 4/group). Vit E = vitamin E *: P<0.05 versus control group #: P<0.05 versus diclofenac 30 mg/kg-only group +: P>0.05 diclofenac 30 mg/kg and vit E versus control

The serum creatinine levels obtained from the normal control rats and DCF (30 mg/kg and 10 mg/kg)-treated rats with or without vitamin E (250 mg/kg) are shown in Figure 1. The serum creatinine level was significantly higher in high-dose DCF-treated rats compared with the control (2.61±0.54 versus 0.80±0.17; P<0.0001; Table 1). Similarly, rats treated with high-dose DCF only had significantly higher creatinine levels than rats treated with high-dose DCF plus vitamin E (250 mg/kg) (P<0.0001). There was no significant difference in serum creatinine levels between the control group and the high-dose DCF plus vitamin E group (P=0.4660; Table 1).

**Table 1:** Level of biochemical parameters among the control and treated groups of rats Control group (distilled water only), DCFH group = diclofenac 30 mg/kg/day, vit E group = vit E 250 mg/kg/day, DCFH plus vit E group = 30 mg/kg and vit E 250 mg/kg orally. DCFL = diclofenac 10 mg/kg/day, DCFL plus vitamin E = diclofenac 10 mg/kg plus vit E 250 mg/kg/day. Values are given as mean ± SD. Subscripts H and L denote high and low doses, respectively. *: Significant difference from the control group at P<0.0001 #: Significant difference from the DCFH group at P<0.0001 +: non-significant difference from the control group at P>0.05

Groups names	Creatinine (mg/dL)	Urea (mg/dL)	Kidney injury molecule-1 (ng/mL)	Tumor necrotic factor-α (pg/mL)	Nuclear factor kappa‑κB (ng/mL)
Control group	0.80±0.17	125.91±19.86	0.72±0.14	233.04±45.26	1.83±1.15
DCF_H_-group	2.61±0.54*	321.00±51.45*	15.88±4.69*	1254.55±106.92*	6.83±1.17*
Vit E-group	0.67±0.26	129.74±15.02	0.95±0.15	222.48±15.18	2.60±.036
DCF_H_ plus vit E	1.23±0.18#+	194.17±7.19#	7.39±2.48#	349.03±135.91#	2.50±0.49#
DCF_L_	0.13±0.45	159.11±33.86	1.64±1.34	343.50±90.67	2.35±1.19
DCF_L _plus vit E	0.90±0.13	142.23±25.46	0.74±0.23	289.58±78.48	1.79±0.07

**Figure 2:**
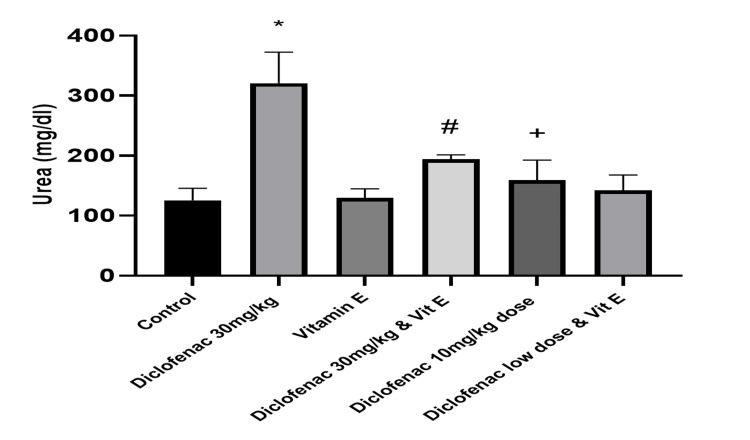
Effect of treatments on the level of serum urea *: P<0.05 versus control group #: P<0.05 versus diclofenac 30 mg/kg-only group +: P>0.05 versus control group

There was a significant increase in the mean value of urea among the high-dose DCF-treated rats compared with the normal control group (321.00±51.45 versus 125.91±19.86; P=<0.0001; Table 1, Figure 2). However, there was no statistically significant difference in the serum urea level between the group administered with a lower dose DCF and the control group. The vitamin E plus high-dose DCF group had a significant decrease in the serum urea level, compared to the high-dose DCF-only group (P=0.0001).

**Figure 3:**
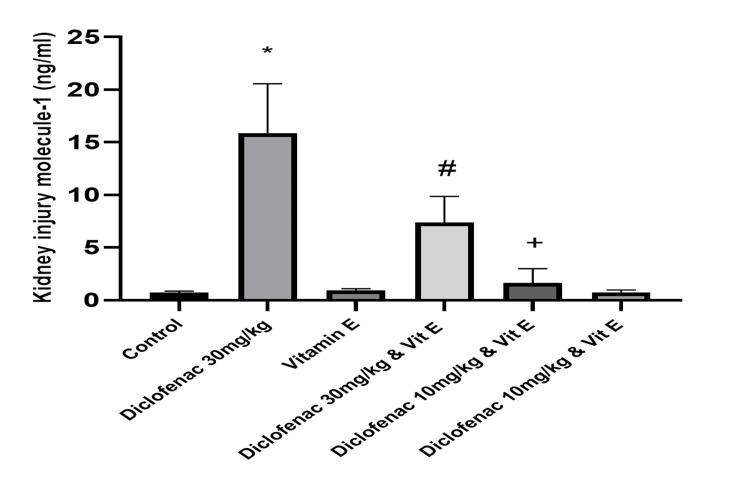
Effect of treatments on the level of kidney injury molecule-1 in renal tissue Data are expressed as mean ± SD. (n = 4/group). Vit E = vitamin E *: P<0.05 versus control group #: P<0.05 versus diclofenac 30 mg/kg-only group +: P>0.05 versus control group

As shown in Figure 3 and Table 1, the level of KIM-1 in the high-dose DCF-only treated group was significantly elevated compared to the control group (P=<0.0001). In contrast, the high-dose DCF co-administered with vitamin E had a significant reduction in the KIM-1 levels compared to the control group (7.39±2.48 versus 0.72±0.14; P=0.0058). There was no observed significant difference in the KIM-1 level between the low-dose DCF group and the control group.

Effect of treatment on markers of inflammation

**Figure 4:**
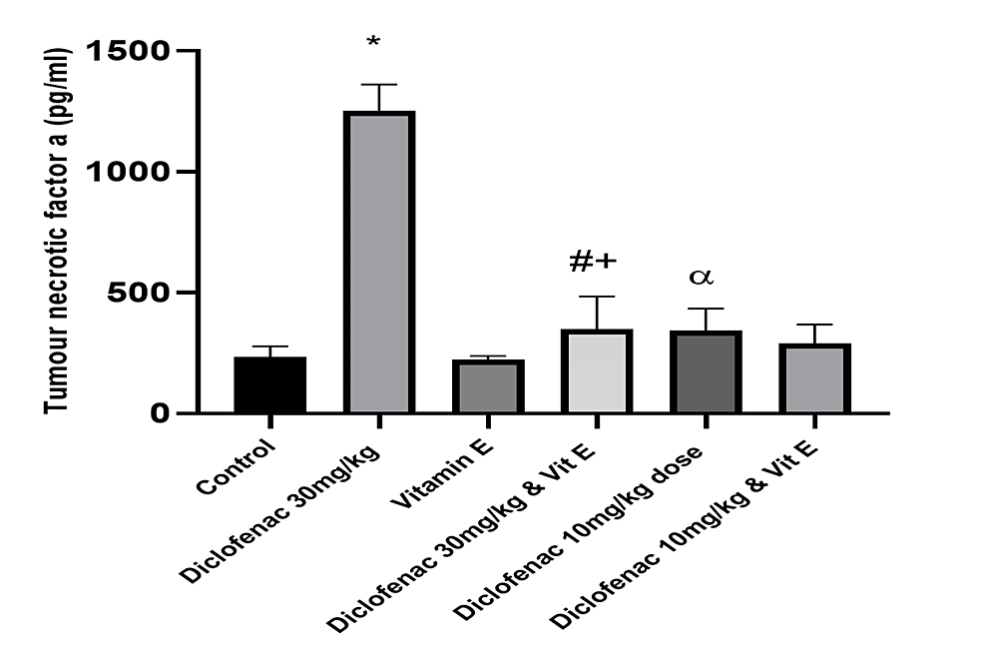
Effect of treatments on the level of tumor necrotic factor-α in renal tissue Data are expressed as mean ± SD. (n = 4/group). Vit E = vitamin E *: P<0.05 versus control group #: P<0.05 versus diclofenac 30 mg/kg-only group α: P<0.05 versus diclofenac 30 mg/kg-only group +: P>0.05 versus control group

**Figure 5:**
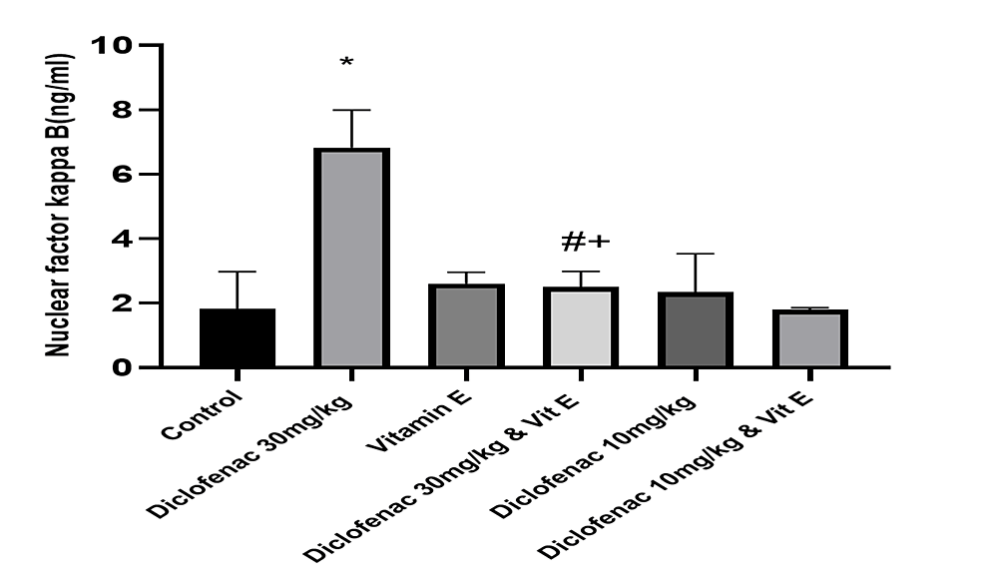
Effect of treatments on the level of nuclear factor kappa-B in renal tissue Data are expressed as mean ± SD. (n = 4/group). Vit E = vitamin E *: P<0.05 versus control group #: P<0.05 versus diclofenac 30 mg/kg group +: P>0.05 versus control group

The renal proinflammatory markers, TNF-α, and the NF‑κB showed a similar trend between the normal control group and the high-dose DCF-treated group. TNF-α and NF-κB levels were significantly increased in the high-dose DCF-treated group compared to the control (1254.55±106.92 versus 233.04±45.26 and 6.83±1.17 versus 1.83±1.15; P<0.0001; Figures 4-5). Vitamin E co-administration with high-dose DCF significantly reduced the levels of the inflammatory marker and NF-κB (Table 1). There was no significant difference in inflammatory marker levels between the low-dose DCF group with or without vitamin E co-administration (Table 1).

Effect of treatment on the level of antioxidants and markers of oxidative stress

**Figure 6:**
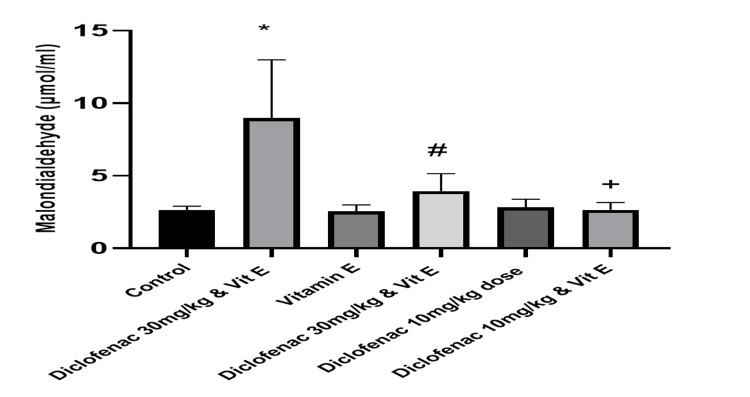
Effect of treatments on the level of malondialdehyde in renal tissue Data are expressed as mean ± SD. (n = 4/group). Vit E = vitamin E *: P<0.05 versus control group #: P<0.05 versus diclofenac 30 mg/kg group +: P>0.05 versus control group

**Figure 7:**
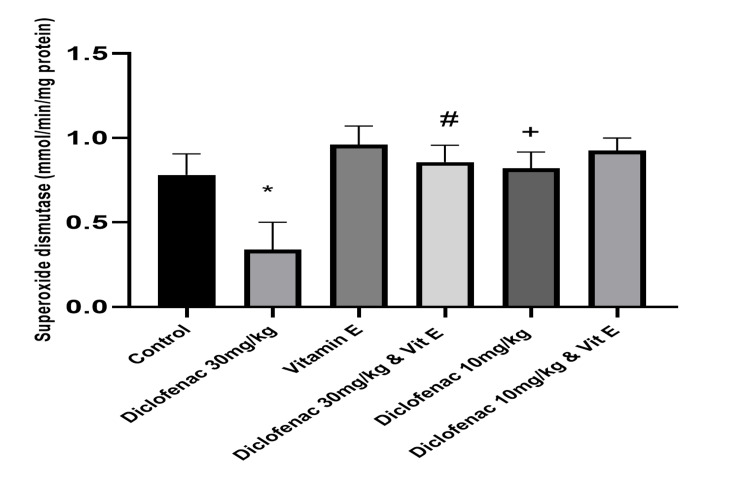
Effect of various treatments on the level of superoxide dismutase in renal tissue Data are expressed as mean ± SD. (n = 4/group). Vit E = vitamin E *: P<0.05 versus control group #: P<0.05 versus diclofenac 30 mg/kg group +: P>0.05 versus control group

**Figure 8:**
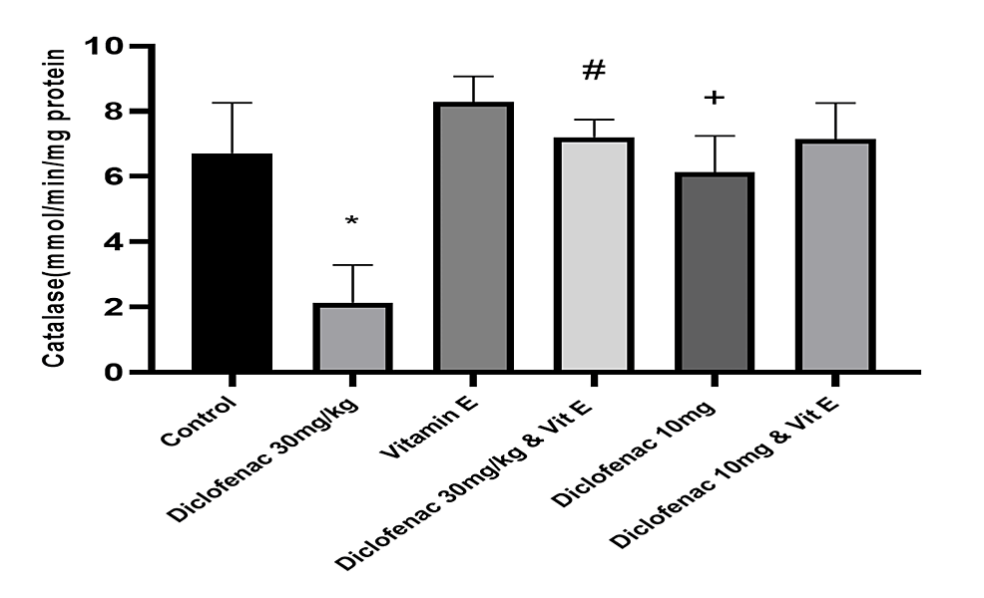
Effect of various treatments on the level of catalase in renal tissue Data are expressed as mean ± SD. (n = 4/group). Vit E = vitamin E *: P<0.05 versus control group #: P<0.05 versus diclofenac 30 mg/kg-only group +: P>0.05 versus control group

**Figure 9:**
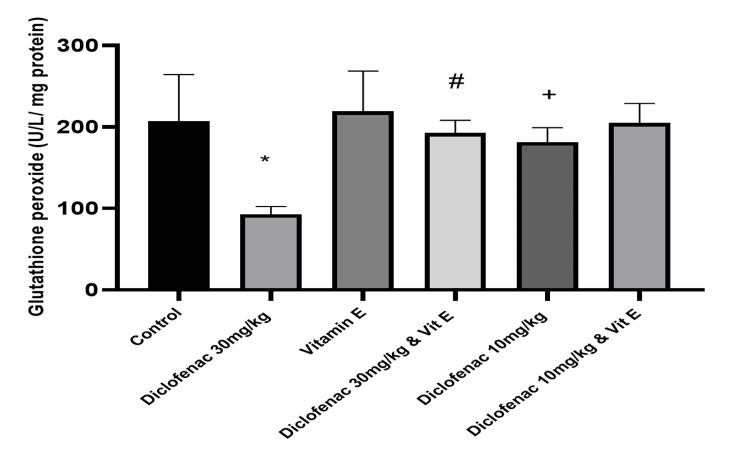
Effect of various treatments on the level of glutathione peroxide in renal tissue Data are expressed as mean ± SD. (n = 4/group). Vit E = vitamin E *: P<0.05 versus control group #: P<0.05 versus diclofenac 30 mg/kg group +: P>0.05 versus control group

Compared to the control group, high-dose diclofenac caused a significant increase in the level of malondialdehyde (2.62±0.29 versus 8.97±4.0183; P<0.00). Superoxide dismutase, catalase, and glutathione peroxide levels were concomitantly reduced (0.78±0.13 versus 0.34±0.16, 6.71±1.55 versus 2.14±1.15, and 206.82±57.67 versus 83.77±7.24, respectively; P=0.0001; Table 2, Figures 6-9). However, in the high-dose DCF plus vitamin E group, there was a statistically significant reduction in lipid peroxidation markers and a concomitant increase in antioxidant enzyme levels in kidney homogenates compared to the control group (Table 2, Figure 6-9). Administration of low-dose diclofenac with or without vitamin E did not significantly change the level of these markers (Table 2).

**Table 2:** Level of renal antioxidants and marker of lipid peroxidation in normal and experimental groups of rats Control group (distilled water only), DCFH group = diclofenac 30 mg/kg/day, vit E group = vit E 250 mg/kg/day, DCFH plus vit E group = 30 mg/kg and vit E 250 mg/kg orally, DCFL= diclofenac 10 mg/kg/day, DCFL plus vit E = diclofenac 10 mg/kg plus vit E 250 mg/kg/day. Values are given as mean ± SD. Subscripts H and L denote high and low doses, respectively. *: Significant difference from the control group at P<0.0001 #: Significant difference from the DCFH group at P<0.0001 +: non-significant difference from the control group at P>0.05

Groups	Malondialdehyde (μmol/ml)	Superoxide dismutase (mmol/min/mg protein)	Catalase (mmol/min/mg protein)	Glutathione peroxidase (U/L/ mg protein)
Control group	2.62±0.29	0.78±0.13	6.71±1.55	206.82±57.67
DCF_H_-group	8.97±4.02*	0.34±0.16*	2.14±1.15*	83.77±7.24*
Vit E-group	2.56±0.44^+^	0.96±0.11	8.26±0.78	219.20±49.40
DCF_H_ plus Vit E	3.94±1.21^#+^	0.85±0.10^#+^	7.21±0.53^#+^	192.89±15.12^+#^
DCF_L_	2.83±0.55^+^	0.82±0.10	6.12±1.12	181.38±17.72^+^
DCF_L _plus Vit E	2.64±0.51	0.92±0.07	7.16±1.01	205.11±23.86

Histopathological findings

**Figure 10:**
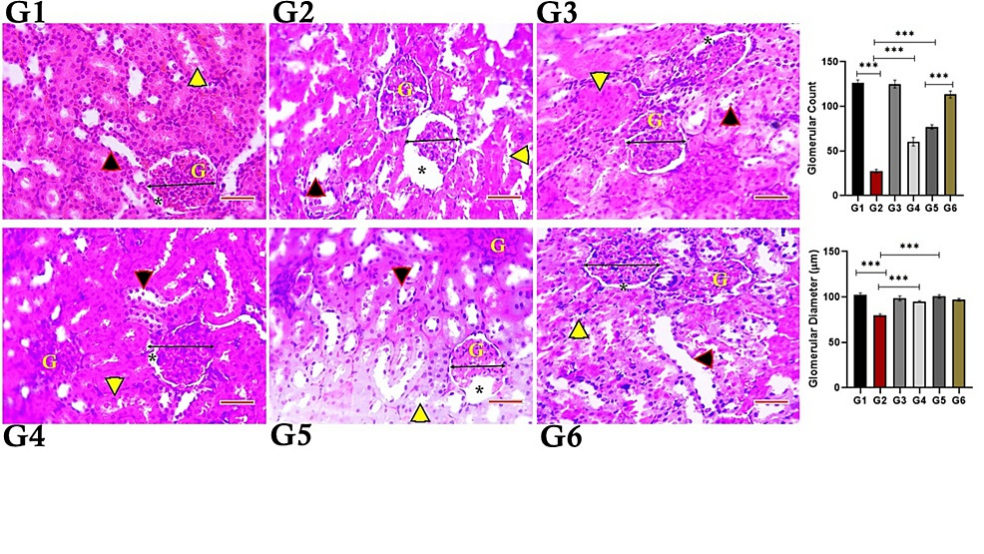
Photomicrographs showing histoarchitecture of the nephron, glomerular count, and diameter in diclofenac with or without vitamin-E-treated rats G1: Control group; revealed normal architecture of the nephron. G2: revealed severe degeneration, and a significant decrease in the glomerular count and diameter. G3: normal architecture of the nephron. G4: revealed significant increase in the glomerular count and diameter when compared to G2. G5: mild degeneration of the nephron, when compared to control. G6: revealed a significant increase glomerular count when compared with the G5 group. (Black arrow-head: indicates distal convoluted tubules, yellow arrow-head: indicates proximal convoluted tubules, Star: urinary space, G: glomerulus). Scale bar: 51 µm

Figure 10 shows that the control group has a normal nephron architecture, while the high-dose diclofenac-only group has severe degeneration and a significant decrease in glomerular count and diameter. However, the high-dose diclofenac plus vitamin E group had a significant increase in glomerular count and diameter compared to the high-dose diclofenac-only group. Low-dose diclofenac treatment with or without vitamin E did not significantly change the glomerular apparatus or tubular integrity.

## Discussion

This study investigated the ameliorating effect of vitamin E in a DCF-induced nephrotoxic rat model. DCF caused significant increases in serum urea and creatinine, renal KIM-1, lipid peroxidation (MDA levels), and inflammatory mediators (TNF-α and NF-κB). Vitamin E treatment significantly elevated the levels of antioxidants GPx, SOD, and CAT in renal tissue, demonstrating its renal protective role. Similarly, histopathological examinations of renal tissue further supported the results of biochemical assays. Additionally, DCF-induced morphological changes in kidney tissues were ameliorated by vitamin E treatment.

The result of this research is similar to previous studies that investigated the nephrotoxic effect of DCF [3,16,17]. DCF-induced oxidative stress in renal tissue may alter the structure and function of mitochondria in renal cells. This may result in altered antioxidant mechanisms and increased production of inflammatory mediators, causing histopathological changes, cellular dysfunction apoptosis of renal tissue, and ultimately nephrotoxicity [10,18,19]. Hickey et al. demonstrated the nephrotoxic effect of DCF. In their study, a fivefold increase in blood urea nitrogen was documented with a very high dose of DCF (300 mg/kg per oral for 24 hours) compared to that of the control [20].

This study is consistent with previous findings that DCF is a strong inducer of oxidative stress, as evidenced by a significant increase in MDA levels [20]. We also observed a dose-dependent increase in MDA levels and a reduction in SOD, CAT, and GPx activity, which was alleviated by vitamin E treatment. Similarly, other studies [2,18] have shown that DCF administration reduces SOD, CAT, and GPx activity and increases H_2_O_2_ and MDA levels in the kidneys of treated animals, suggesting that DCF may impair kidney function through oxidative stress [4].

In a comparative investigation conducted by Ajith et al. [4], the potential nephroprotective effects of vitamin C (ascorbic acid) and vitamin E (alpha-tocopherol) were explored on cisplatin-induced oxidative renal damage in mice. The results of the study indicated that both vitamins effectively mitigated the nephrotoxicity induced by cisplatin. Furthermore, their research demonstrated a decrease in renal antioxidant enzyme levels following cisplatin induction, which was subsequently elevated in the groups treated with the vitamins. In conclusion, the study suggested that higher doses of vitamin C and vitamin E offer more substantial benefits in preventing lipid peroxidation and the depletion of renal antioxidants. Comparable findings were documented by El-Shafei et al., supporting the important role of vitamin E in reducing oxidative stress and kidney damage induced by DCF sodium [21].

In this current study, analysis of the inflammatory markers demonstrated that administration of DCF increases TNF-α and nuclear factor kappa light chain enhancer of activated B cell proteins expression in kidney tissues. However, vitamin E administration suppressed elevated inflammatory cytokines, suggesting that inhibition of the TNF-α/NF-κB pathway may be one of the possible mechanisms of vitamin E's renal protective effect.

Evidence suggests that inflammation is closely associated with the pathogenesis of nephrotoxicity [3,16,18]. Cytokines such as TNF-α, IL-6, IL-1β, and NF‑κB were identified to be upregulated in DCF-induced nephrotoxicity [16,18]. In experimentally induced immune-mediated glomerulonephritis, inflammation has been shown to play an important pathogenic role [22]. A multicentre cohort study of 98 patients with acute renal failure found significantly higher plasma levels of all measured cytokines compared with healthy subjects, and higher cytokine levels were associated with death [23].

An antioxidant flavonoid, fisetin, has been shown to protect the kidneys of experimental mice from damage by inhibiting inflammation pathways (the steroid receptor coactivator, Src-mediated NF-κB, and MAPK signaling), which leads to lower levels of serum creatinine, blood urea nitrogen, and KIM-1 [24].

The pathophysiology of DCF-induced nephrotoxicity may involve multiple factors and numerous signaling pathways. However, the role of oxidative stress, particularly the production of reactive oxygen species (ROS) and antioxidant system dysfunction, is well-established [2,9,17,18,25]. DCF mainly produces ROS by targeting microsomes and mitochondria. Mitochondria are essential organelles in the kidney they produce cellular energy for metabolic processes.

During mitochondrial metabolism, ROS are produced. These ROS function as secondary messengers, inducing [26] post-translational modifications (PTM) in proteins and activating or deactivating different cell signaling pathways. However, in the presence of nephrotoxic substances, ROS overproduction can lead to oxidative stress, inducing dysregulation of redox-sensitive signaling pathways, mitochondrial dysfunction, and altered metabolism [5,27]. These imbalances can ultimately lead to changes in the cell redox-sensitive signaling pathways, causing inflammation and apoptosis cell death [3]. Other targets of ROS attack include lipid proteins, leading to increased malondialdehyde and decreased enzymatic and non-enzymatic antioxidant activities (GPx, SOD, CAT, and glutathione) [20,25,28,29].

This study found that DCF treatment decreased the activities of SOD, CAT, and GPx in rat kidneys. El-Maddawy et al. found similar results in experimental rats treated with low and high doses of DCF [30]. The level of lipid peroxidation marker MDA was also elevated in this study, consistent with the findings of other studies.

This study found that co-administration of vitamin E restored the reduced GPx levels in DCF-treated rats. The possible mechanisms by which DCF depletes GPx activity in rats include conjugation with DCF, rapid utilization by glutathione-dependent enzymes, and overutilization by the cell to combat ROS generation.

## Conclusions

This study highlights the potential nephrotoxicity associated with DCF, particularly when used at a certain dosage over an extended period. The observed significant increases in serum urea, creatinine, KIM-1, TNF-α, NF-κB, and malondialdehyde levels following DCF administration underscore the renal damage induced by the drug. However, co-administration of vitamin E with DCF demonstrated a substantial nephroprotective effect. The significant reduction in pro-inflammatory cytokines and malondialdehyde levels, coupled with the amelioration of renal function indices, oxidative stress parameters, and the observed histopathological improvements, collectively highlight the potential therapeutic benefits of vitamin E in mitigating DCF-induced nephrotoxicity.

This research underscores the significance of exploring adjunct therapies such as vitamin E to counteract the renal adverse effects of DCF, especially those on prolonged treatment regimens or with background renal disease, which can ultimately improve patient safety and treatment outcomes in clinical practice. Further investigations are needed to establish appropriate dosage regimens for the clinical application of vitamin E in conjunction with DCF therapy.
